# Proteases and HPV-Induced Carcinogenesis

**DOI:** 10.3390/cancers14133038

**Published:** 2022-06-21

**Authors:** Gabriel Viliod Vieira, Fernanda Somera dos Santos, Ana Paula Lepique, Carol Kobori da Fonseca, Lara Maria Alencar Ramos Innocentini, Paulo Henrique Braz-Silva, Silvana Maria Quintana, Katiuchia Uzzun Sales

**Affiliations:** 1 Department of Cell and Molecular Biology and Pathogenic Bioagents, Ribeirao Preto Medical School, University of Sao Paulo, Ribeirao Preto 14049-900, SP, Brazil; gabrielviliod@gmail.com (G.V.V.); kobori@usp.br (C.K.d.F.); lara.m.alencar@hotmail.com (L.M.A.R.I.); 2 Department of Gynecology and Obstetrics, Ribeirao Preto Medical School, University of Sao Paulo, Ribeirao Preto 14049-900, SP, Brazil; fernanda.somera.santos@gmail.com (F.S.d.S.); quintana@fmrp.usp.br (S.M.Q.); 3 Department of Immunology, Biomedical Sciences Institute, University of Sao Paulo, Sao Paulo 05508-000, SP, Brazil; alepique@icb.usp.br; 4 Clinical Hospital of Ribeirao Preto Medical School, University of São Paulo, Ribeirao Preto 14049-900, SP, Brazil; 5 Department of Stomatology, School of Dentistry, University of Sao Paulo, São Paulo 05508-000, SP, Brazil; pbraz@usp.br; 6 Laboratory of Virology, Institute of Tropical Medicine of Sao Paulo, School of Medicine, University of Sao Paulo, Sao Paulo 05403-000, SP, Brazil

**Keywords:** HPV, carcinogenesis, proteases, cervical cancer, anogenital cancer, oropharyngeal cancer

## Abstract

**Simple Summary:**

Human papillomavirus (HPV) infection is a sexually transmitted disease with high prevalence worldwide. Although most HPV infections do not lead to cancer, some HPV types are correlated with the majority of cervical cancers, and with some anogenital and oropharyngeal cancers. Moreover, enzymes known as proteases play an essential role in the pathogenic process in HPV-induced carcinogenesis. This review highlights the role of proteases and recent epidemiological data regarding HPV-dependent carcinogenesis.

**Abstract:**

Persistent infection with Human papillomavirus (HPV) is the main etiologic factor for pre-malignant and malignant cervical lesions. Moreover, HPV is also associated with oropharynx and other anogenital carcinomas. Cancer-causing HPV viruses classified as group 1 carcinogens include 12 HPV types, with HPV 16 and 18 being the most prevalent. High-risk HPVs express two oncoproteins, E6 and E7, the products of which are responsible for the inhibition of p53 and pRB proteins, respectively, in human keratinocytes and cellular immortalization. p53 and pRB are pleiotropic proteins that regulate the activity of several signaling pathways and gene expression. Among the important factors that are augmented in HPV-mediated carcinogenesis, proteases not only control processes involved in cellular carcinogenesis but also control the microenvironment. For instance, genetic polymorphisms of matrix metalloproteinase 1 (MMP-1) are associated with carcinoma invasiveness. Similarly, the serine protease inhibitors hepatocyte growth factor activator inhibitor-1 (HAI-1) and -2 (HAI-2) have been identified as prognostic markers for HPV-dependent cervical carcinomas. This review highlights the most crucial mechanisms involved in HPV-dependent carcinogenesis, and includes a section on the proteolytic cascades that are important for the progression of this disease and their impact on patient health, treatment, and survival.

## 1. Introduction

Viral infection in humans leads to a wide variety of diseases, such as smallpox, polio, and measles [1]. Some have caused recent significant pandemics, such as H1N1 influenza in 2009 and the SARS-CoV-2 coronavirus in 2020 [2,3]. Some viruses can increase the host cell’s lifespan and deregulate critical signaling pathways through the activation of oncogenes and/or the suppression of tumor suppressor genes [4]. Such viruses are classified as carcinogenic to humans (group 1) by the International Agency for Research on Cancer (IARC) of the World Health Organization (WHO) [5,6]. At least seven viruses are related to cancer development in humans: Epstein–Barr virus (EBV), hepatitis B virus (HBV), hepatitis C virus (HCV), human papillomavirus (HPV), human T-cell lymphotropic virus (HTLV-1), Kaposi’s sarcoma-associated herpesvirus (KSHV), and Merkel cell polyomavirus (MCV or MCPyV); they contribute to 10–15% of cancers worldwide [5,7,8]. These viruses, known as tumor viruses, induce changes in cellular functions that ultimately lead to cancer development [4,9].

The transformation of a healthy cell into a tumor cell is a complex, multi-step process [4]. During the carcinogenic process, the malignant cell suffers genetic and epigenetic modifications that are selected and expressed as capabilities known as the hallmarks of cancer: genome instability and mutation, resistance to cell death, the deregulation of cellular energetics, sustained proliferative signaling, the evasion of growth suppressors, the avoidance of immune destruction, the enabling of replicative immortality, tumor-promoting inflammation, and the activation of invasion and metastasis, inducing angiogenesis [9,10,11,12].

Although oncoviruses can participate in oncogenesis, they are not sufficient for the development of cancer, and inflammation, host immune response and environmental conditions are also involved in this process [4,5,10,13].

Papillomaviruses are epitheliotropic, small, double-stranded DNA viruses that infect the mucosa or the skin of many animals’ species mucosa [14,15]. Although more than 200 genotypes can infect humans, only 12 HPV genotypes with carcinogenic properties—classified as group 1 carcinogens by the International Agency for Research on Cancer (IARC)—are known [6,16,17]. Mucosal transmission occurs mainly by sexual contact [17,18]. However, other transmission routes are also known [18]. Studies have demonstrated the vertical transmission of HPV from mother to fetus, as well as the presence of HPV viral DNA in breast milk, amniotic fluid, the umbilical cord, and the placenta [19,20,21]. Newborns can also become infected through skin-to-skin contact with other relatives, as well as oral lesions related to HPV infection, such as oral squamous papilloma, condyloma acuminatum, verruca vulgaris, and multifocal epithelial hyperplasia; genital HPV infection in children is a warning sign for sexual abuse [22,23,24,25].

HPV viruses are divided into five genera according to the sequence of their genotype, known as α, β, γ, μ, and ν [26]. The HPV alpha and gamma groups infect skin and mucosal tissue, whereas the beta-, nu- and mu-subtypes infect cutaneous sites, even without clinical manifestations [27,28]. All of the 12 HPV genotypes that are classified as group 1 carcinogens belong to the alpha genus: HPV16, 18, 31, 33, 35, 39, 45, 51, 52, 56, 58, and 59 [29]. Indeed, alpha-HPVs are transmitted through sexual contact, and can be considered the leading group of causative agents of sexually transmitted infections globally [28]. Moreover, their incidence rises sharply after the first sexual intercourse [30]. As the natural immune response to this virus is weak and variable, one person may acquire different types of HPV infections [14]. Low-risk alpha HPV types causing benign genital warts or condylomata acuminata, as well as common and plantar warts, are also found [29].

HPV infection prevalence can also vary widely due to regional differences and age groups. Cervical infections are usually asymptomatic and transient, with 70–90% of infections resolving within 1–2 years [15]. Younger women, especially those under 25, are the most affected by HPV infections [31]. Nevertheless, most of these infections are cleared by the immune system and have no clinical manifestation [15,16]. In order to help understand the impact that HPV has in cancer development, we analyzed the key statistics of HPV-related cancers in the world in Table 1 (adapted from [32]).

## 2. HPV Carcinogenesis

HPV infection has been linked to several malignancies, such as cervical carcinoma, female and male anogenital carcinomas (vulvar, vaginal, anal, and penile), and head and neck squamous cell carcinomas (HNSCCs) [14]. In these anatomical sites, however, the behavior of HPV is less understood, as the prevalence is lower than that of cervical carcinomas [14].

According to the type of lesion they generate, they are subdivided into low-risk HPVs and high-risk HPVs [28]. Low-risk HPVs are associated with the development of warts and benign lesions, while high-risk HPVs are associated with precancerous and cancerous lesions [33]. HPV16 and HPV18 stand out for their greater capacity to lead to cancer development; they account for approximately 60% and 15% of cases of invasive cervical cancer worldwide, respectively [14].

The HPV genome can be divided into two types of genes: (i) early genes (from E1 to E7), which are responsible for viral genome gene expression and replication, and which also modulate host cell proliferation and differentiation [34,35], and (ii) late genes (L1 and L2) which are responsible for the formation of viral capsid [36,37,38]. During infection, the viral genome may integrate into the host cell genome. When found in cancer cells, which occurs in most cases, the viral genome is disrupted in the E1/E2 region. E2 is a transcription factor that binds to the HPV LCR (Long Control Region) and maintains the weak transcriptional activity of the promoter. If E2 expression is lost, other transcription factors bind to the LCR and increase the expression of E6 and E7, which are two bona fide oncogenes present in the HPV genome. This event is important for cellular immortalization and transformation by HPV [39,40,41]. Persistent infection is a major risk factor that increase HPV genome integration.

Molecular events caused by infection and, in some cases, cellular transformation will cause lesions classified histopathologically as cervical intraepithelial neoplasia (CIN) and further to cancer, which is then sub-classified as CIN 1—mild dysplasia, CIN 2—moderate dysplasia, or CIN 3—severe dysplasia to carcinoma in situ [42,43].

The main orchestrators of cellular transformation by HPV are the oncoproteins E6 and E7. They inactivate p53 and retinoblastoma protein (pRB), respectively, leading to the cell’s inability to control the cell cycle checkpoints correctly, and thus exacerbating cell proliferation [40]. Significantly, the E7-dependent inhibition of pRB leads to the cell cycle S phase transition, promoting cell proliferation and viral transcription [44,45]. Another critical aspect of E7 is that it binds to p21 and p27, which are proteins belonging to the cyclin-dependent kinase (CDK) interacting protein/kinase inhibitory protein (CIP/KIP), and are involved in regulating the cell cycle, which increases cyclin-dependent kinase 2 (CDK2) activity, collaborating with the cell entering the G1 to S phase [46,47]. In a normal physiological condition, p53 would counteract the effects of exacerbated cell proliferation while activating the cellular DNA damage response (DDR) during viral DNA integration into the host genome, leading to the inhibition of cell growth and apoptosis [48,49]. However, HPV E6 inactivates p53 by targeting its proteasomal degradation and forming a complex with the E3 ubiquitin-protein ligase E6-associated protein, E6AP [50,51]. Moreover, it is also important to highlight the fact that high-risk E6 has been reported to bind the hTERT protein as well as the repeating DNA sequence of telomeric DNA, in addition to controlling telomerase activity [52]. High-risk E6′s role in hTERT, telomerase, and telomeric DNA is thus multilayered, emphasizing its crucial and overlapping role in immortalization [53].

Cells infected with HPV are able to stop infection by activating signaling pathways that result in the induction of anti-viral status and IFN type I secretion. However, both E6 and E7 display mechanisms of suppressing this response. Among other effects, E6 binds to IRF-3 (Interferon Response Factor-3), inhibiting its activity. E7 binds to IRF-1 and recruits HDAC (histone deacetylase) to the promoters that would be activated by IRF-1 but are suppressed due to E7 activity [54]. Moreover, E6 and E7 can inhibit the STAT-1 and protein kinase R (PKR) pathways in infected cells [55].

**Figure 1:**
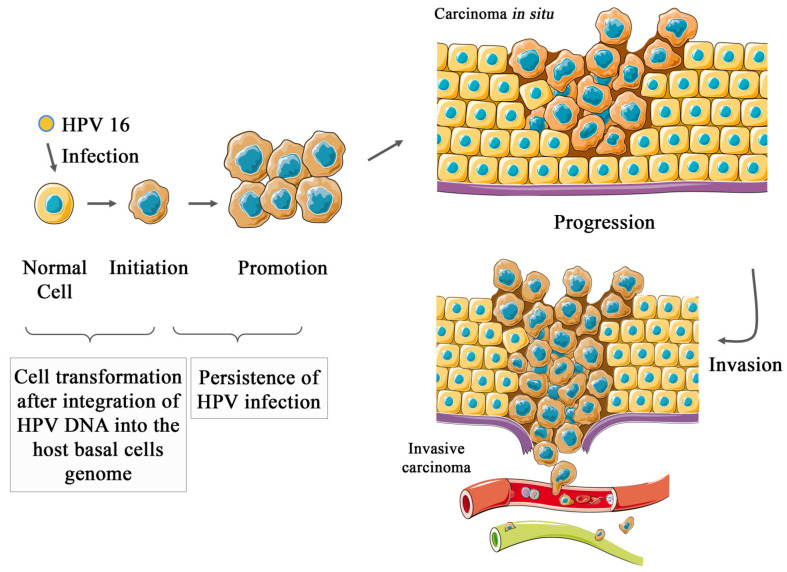
HPV carcinogenesis. This figure shows the general mechanisms of HPV carcinogenesis. After HPV infection, especially the high-risk types (e.g., HPV16), the oncogenes of the HPV can integrate into the host basal cell genome, if the infection is recurrent. If this step happens, it is possible to observe the initiation of the tumor, which can progress to the promotion stage, in which many cells transform to cancer cells. After this, the progression of the cancer cells takes place, and the carcinoma develops in situ and can progress to the invasion step, in which the cancer cells migrate to other tissues, leading to metastasis. Parts of the figure were drawn using pictures from Servier Medical Art. Servier Medical Art by Servier is licensed under a Creative Commons Attribution 3.0 Unported License (https://creativecommons.org/licenses/by/3.0/ accessed on 15 April 2022).

The adaptive immune system develops a natural response to HPV infections, and the prevalence of viral infection for long enough to lead to malignant cell transformation depends on the interaction of host, virus, and behavioral variables. As of yet, there is no way to predict who will develop cancer and who will clear the HPV infection [14]. However, most cases regress spontaneously [14]. The general mechanisms of HPV carcinogenesis are illustrated in Figure 1 (the figure was partly generated using Servier Medical Art, provided by Servier, licensed under a Creative Commons Attribution 3.0 Unported License).

In order to assess the immune system’s role in HPV-induced carcinomas, many investigators have studied the behavior of HPV infections in the HIV-infected population [56,57]. Because HIV leads to acquired immunodeficiency, the association of HPV-induced cancers with HIV-infected patients shows the input of a deficient immune system towards the development of carcinomas [58]. Moreover, the prevalence of invasive cervical carcinoma among women with HIV infection is higher than that in the general population [56]. This association was well characterized, and in 1993 invasive cervical carcinoma was included as an AIDS-defining event and, therefore, a hallmark of immunodeficiency [58]. Guiguet and collaborators demonstrated a significant association between immunodeficiency and anal and cervical carcinomas. The duration of immunodeficiency and viral replication appears to play an essential role in developing anal cancer, as the CD4 cell count and antiretroviral therapy have been associated with anal cancer [59].

## 3. Proteases and HPV Carcinogenesis

Proteases, also known as peptide hydrolases, are found in all organisms (from viruses to vertebrates), and are classified as enzymes that can cleave peptide bonds [60,61,62,63]. There are more than 400 proteases described in humans, and more than 14% have the potential to serve as drug targets for a variety of diseases [64,65].

A critical aspect of the proteases is substrate specificity. Some proteases, such as trypsin, display broad specificity and are capable of cleaving many different substrates [66,67,68,69]. Other proteases, such as the urokinase-type plasminogen activator (uPA), are selective and cleave a limited number of substrates [63,69].

Proteases can be subdivided into two major groups: exopeptidases and endopeptidases [70]. Exopeptidases are known to hydrolyze the substrate chain’s amino or carboxy terminal, and the conferred specificity is determined by the fragment size [71,72]. Endopeptidases are proteases that can cleave the amino acids that are non-terminal, and the classification relies on the chemical group present in the catalytic domain. Six classes of endopeptidases have been described: (i) cysteine proteases, (ii) aspartic acid proteases, (iii) threonine proteases, (iv) glutamic acid proteases, (v) serine proteases, and (vi) metalloproteases [73].

Proteases are known to participate in different physiological processes, from the degradation of proteins for recycling, to apoptosis, the cell cycle, skin desquamation, semen liquefaction, epithelial differentiation, the regulation of blood pressure, and homeostasis [74,75,76,77]. It is essential to highlight the facts that proteases are synthesized as inactive zymogens, and that they have to be cleaved to be activated, which can occur irreversibly through post-translational modifications, co-factors ligation, and changes in pH, among others [77]. Therefore, the activation of proteases is a very regulated process that prevents the uncontrolled activation of the enzymes in the cell [73,78]. The dysregulation of some proteases’ expression can lead to the development of diseases, such as cancer. These enzymes are also involved in the degradation of the extracellular matrix and the activation of growth factors and pro-inflammatory mediators, which participate in malignant transformation and tumor progression [79].

Serine proteases are a family of proteases characterized by the amino acids responsible for the catalytic activity in proteolysis, which are serine, aspartate, and histidine [80]. This family of proteases is involved in different biological processes, such as epithelial barrier formation, skin desquamation, fertilization, embryonal development, cell signaling, and tissue morphogenesis [81].

As part of this family, there is a subgroup composed of membrane-anchored serine proteases, which is divided into (i) GPI—serine proteases that are anchored in the plasma membrane by a glycosylphosphatidylinositol anchor, (ii) Type I—serine proteases that have a single pass domain in the plasma membrane located close to the C-terminus end, and (iii) Type II—serine proteases that are anchored in the plasma membrane and have an anchor sign located close to the N-terminus end [77,82].

Matriptase, a type II transmembrane serine protease, is expressed in different epithelial tissues, such as the skin, gastrointestinal tract, lungs, kidneys, prostate, and mammary glands [83,84]. This protease is responsible for activating uPA (urokinase plasminogen activator), which is related to cell adhesion and migration regulation, and the activation of growth factors and metalloproteases zymogens [85,86]. Different studies have shown that when matriptase is less expressed, there is also less activation of uPA in the cells of ovary and prostate cancer [87,88]. Matriptase is also responsible for activating the PI3K-Akt-mTOR pathway after the proteolytic activation of the hepatocyte growth factor precursor (pro-HGF), which can promote cell proliferation and decrease apoptosis [82]. PAR-2 (protease-activated receptor 2), a receptor expressed in different cell types, is related to cell adhesion, the maintenance of the skin barrier, and inflammatory responses [89,90]. One study has shown that the absence of PAR-2 inhibited the appearance of premalignant lesions and spontaneous or induced carcinogenesis in mice that overexpress matriptase in the basal layer of the epithelia, which highlights the importance of PAR-2 activation by matriptase in oncogenesis [90]. It has already been described that matriptase is dysregulated in different types of epithelial cancers and, more specifically, carcinomas [84,90,91,92,93,94,95].

Furthermore, matriptase is inhibited by the hepatocyte growth factor activator inhibitor-1 and -2 (HAI-1 and HAI-2), which are serine proteases inhibitors [96,97]. HAI-1 is a type I transmembrane serine protease inhibitor encoded by the SPINT1 gene. One study has shown that HAI-1 is a potent inhibitor of hepsin, matriptase, and prostasin in HPV-positive cells (SiHa and HeLa). In cervical tissue analysis, HAI-1 expression was correlated with higher rates of tumor growth, the stage of the disease, stromal invasion, vaginal invasion, and lymph node metastasis [98]. Moreover, patients exhibiting higher levels of HAI-1 exhibited decreased disease-free and overall survival [98]. Similarly, analyzing cervical cancer specimens and the biological functions of HPV-positive cell lines, findings have indicated that a lower expression of HAI-2 in cervical cancer may be correlated with poor prognosis as well [99].

Other subgroups of serine proteases are the ones that are secreted to the extracellular space, such as the kallikreins. Kallikreins are serine proteases that can be divided into (i) plasma kallikrein, with one member, KLKB1; and (ii) tissue kallikreins, with fifteen members, which have either tryptic or chymotryptic specificity [100]. KLK proteases are found in almost every tissue, with different physiological functions, such as skin desquamation and seminal clot liquefaction, related with various cancers, Parkinson’s and Alzheimer’s diseases [100]. The relationship between KLKs’ activation and different types of viruses, such as influenza and HPV, has been described [101,102]. After HPV infection, the virus has to remove the capsid to expose the viral genome. The HPV16 virus can bind to heparan sulfate proteoglycans located in the host cell surface or the ECM [103,104,105]. After the binding, the late gene 1 (L1) undergoes a conformational change, which leads the protein to be cleaved by Kallikrein 8 (KLK8) [102]. Furthermore, the knockdown of KLK8 in HeLa and HaCaT cell lines has shown an inhibitory effect of HPV16 infection, while the irreversible serine protease inhibitor AEBSF [4-(2-aminoethyl) benzenesulfonyl fluoride] also had the same effect [106]. The conformational change caused after L1 cleavage by KLK8 facilitates access to late gene 2 (L2) protein, which is found in the capsid lumen and facilitates the uncoating of the virus [106]. In another study, using liquid chromatography-tandem mass spectrometry (LC-MS/MS) in cervical tissues, the authors found that 95 proteins were dysregulated in the samples. Among those, the expression of ECM2 and the serine proteases KLK6 and MASP1 were increased in a stage-dependent manner. In particular, KLK6 was considered a highly significant prognostic marker, as it demonstrated a decrease in the overall survival (OS) and disease-free survival rates, showing that this protease may be considered a potential biomarker for the diagnosis and prognosis of cervical cancer [107].

Metalloproteases (MMPs) are critical enzymes which are responsible for the degradation of the extracellular matrix (ECM) [108]. These proteases participate in various physiological processes, especially in connective tissue remodeling, such as the postpartum involution of the uterus, ovulation, and wound healing, and in pathological processes such as joint destruction in rheumatoid diseases [109,110]. It is known that metalloproteases are secreted as zymogens, and that they are activated proteolytically. Metalloproteases are divided into their respective subfamilies: collagenases (MMP-1, MMP-8, MMP-13), gelatinases (MMP-2 and MMP-9), stromelysins (MMP-3, MMP-10, MMP-11, and MMP-17), matrilysins (MMP-7 and MMP-26), membrane types (MMP-14, MMP-15, MMP-16, MMP-24, and MMP-25), and other types (MMP-12, MMP-19, MMP-20, MMP-21, MMP-22, MMP-28, and MMP29) [72,111].

Studies of cervical tissues with cervical intraepithelial neoplasia and invasive squamous cell carcinomas (tested for HPV expression) have shown that the expression of MMP-2 in preinvasive lesions and MMP-1 and MMP-2 in invasive cancer suggests a gradual increase in the potential of cancer invasion [108]. Furthermore, analyses of the expression of MMP-1 in cell lines (transformed with HPV18) and in clinical samples of cervical squamous cell carcinomas (SCC) have shown that MMP-1 is more expressed in SCC samples when compared with normal tissues, and that this protein can serve as a marker of the invasiveness of SCC [112]. The production of MMP-9 was also up-regulated in cervical intraepithelial neoplasia (CIN 2 and 3) and in invasive carcinomas, which suggests a possible marker for early tumor progression [113].

The E6 HPV viral oncoprotein can interact with PDZ domains, which are 80–110 residue-containing domains that are part of the signaling proteins’ C-terminal [114,115]. The serine protease HTRA-1, which contains a PDZ domain in its C-terminal region, is expressed in various tissues, and is also associated with different pathologies, such as some types of cancer [116,117,118,119]. One study has shown that the overexpression of the serine protease HTRA-1 is responsible for the prevention of cell proliferation in cervical HPV-negative cell lines and increasing cell proliferation in cervical HPV-positive cells, inferring that HTRA-1/E6 interaction is the underlying mechanism for the bypassing of growth arrest in HPV-positive cervical cancer cell lines [120].

Another important group of proteases that are related with HPV carcinogenesis are the Ubiquitin proteases. USP46 is recruited to deubiquitinate and stabilize Cdt2/DTL by the E6 of high-risk HPV but not low-risk HPV [121]. Cdt2—a component of the CRL4Cdt2 E3 ubiquitin ligase—is stabilized, which restricts the amount of Set8, an epigenetic writer, and promotes cell proliferation [121]. USP46 is required for HPV-transformed cells to proliferate, but not for non-HPV cells to proliferate [121]. Human cervical malignancies have a high level of Cdt2, and knocking down USP46 in xenografts stops HPV-transformed tumor growth [121]. Oncogenic E6 recruits a cellular deubiquitinase to stabilize critical cellular proteins, and because the E6-USP46-Cdt2-Set8 pathway is important in HPV-induced malignancies, USP46 is a target for cancer therapy [121]. Another study has shown that USP13 is essential for HPV-positive cervical cancer cells to proliferate, at least in part by deubiquitinating and stabilizing the prosurvival protein Mcl-1 [122]. Importantly, the pharmacological inhibition of USP13 sensitizes HPV-positive cervical cancer cells to BH3 mimetic inhibitors, implying that targeting USP13 could be beneficial in the treatment of these tumors [122]. Furthermore, another study discovered ubiquitin-specific protease 15 (USP15) as an HPV16 E6-interacting protein using the yeast two-hybrid technique [123]. HPV16 E6 polyubiquitin chains and/or ubiquitin precursors are cleaved by USP15, and could boost HPV16 E6 levels by preventing E6 degradation [123]. The degradation of HPV16 E6 was reduced by USP15 in a dose-dependent manner; these findings imply that USP15, as a deubiquitinating enzyme, can stabilize E6 and, as an oncoprotein, can influence biological activities in infected human cells [123]. Besides this, an important study highlighted that long-term hypoxia activates NF-κB, which is mediated via an effect of the HPV-encoded E6 protein on polyubiquitination and the subsequent degradation of the CYLD K63 deubiquitinase in HPV-positive cancer cells [124].

## 4. HPV and the Microenvironment

The carcinogenic process involves more than the HPV-infected cells. Other cells in the microenvironment can influence the cellular fate. As mentioned before, the immune system can eliminate precursor lesions. It has been shown that HPV-infected asymptomatic women display T cell responses against HP, while patients with cancer display regulatory T cell responses toward HPV [125].

Myeloid cells can also display a role in HPV-triggered carcinogenesis. Several groups have shown that as lesions progress from low to high grade to cancer, there is also an increase in the frequency of infiltrating macrophages in the cervical HPV-associated lesions [126,127]. Macrophages display a pro-tumoral role by inhibiting anti-HPV T cell responses [128] in an IL-10-dependent mechanism, and by secreting MMP-9, which—as mentioned before—can promote angiogenesis [129]. Interestingly, the depletion of macrophages did not impair the carcinogenic process in the K14-HPV16 transgenic mouse model, as neutrophils were recruited to the lesions to provide compensatory mechanisms including MMP-9 secretion [130]. Other cells that seem to contribute with HPV-induced carcinogens are the mast cells. These cells secrete several factors, among them tryptase, an enzyme that may stimulate neoangiogenesis and activates PAR2, with the effects described before. It has been observed that the number of mast cells increases in proportion to the cervical lesion grade [131]. There are other groups, however, that have not found the same correlation, concluding that mast cells may display an important role in inflammatory lesions but not neoplastic lesions [132]. Whether the inflammatory response can be part of the carcinogenic process initiated by HPV it is a frequent and much-discussed topic that still needs to be addressed.

HPV-transformed cells activate the transcription factor NFκB (Nuclear Factor kappa B) that activates the transcription of IL-6 and IL-8. IL-6 activates its receptor and the JAK2/STAT3 pathway, which can lead—in the tumor cells—to increased proliferation, survival and epithelial-mesenchymal transition [133]. IL-6 and G-CSF, also secreted by HPV-transformed cells, can activate STAT3 systemically, promoting the accumulation of myeloid cells, including neutrophils, which can then be recruited to the tumor microenvironment by chemotactic molecules, such as IL-8 [134,135].

**Figure 2:**
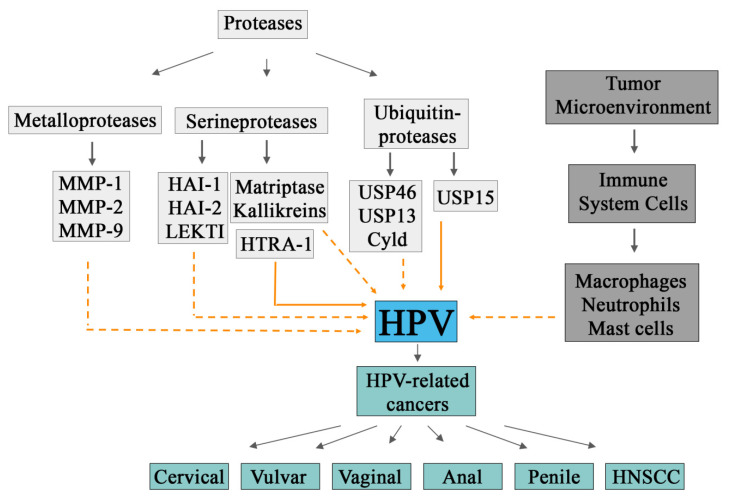
Proteases and the tumor microenvironment interact with HPV. This figure sums up the direct and indirect interactions between proteases, the tumor microenvironment and HPV. Proteases—such as metalloproteases, serine proteases and ubiquitin proteases—participate in HPV carcinogenesis, indirectly (represented by the dashed orange arrow) or directly (represented by the solid orange arrows). The solid gray arrows only indicate the members of each category. Furthermore, the tumor microenvironment is essential for the carcinogenesis of HPV, where the cells from the immune system might participate in that context. The interactions highlighted in this figure can result in the development of different HPV-related cancers, such as cervical, vulvar, vaginal, anal, penile, and head and neck squamous cell carcinomas.

Figure 2 sums up the interactions that might occur between proteases, the tumor microenvironment, and HPV.

## 5. HPV and Cervical Intraepithelial Lesions

Understanding the embryological origin, anatomy, and histology of the uterine cervix is fundamental to understanding the pathogenesis of cervical lesions triggered by HPV infection [136]. The cervix has two distinct portions. The inner portion, called the endocervix, originates from the endoderm and is lined with glandular epithelium; the external portion, called the ectocervix, originates from the ectoderm and consists of non-keratinized stratified squamous cell epithelium. The point of union of these epithelia is called the squamocolumnar junction (SCJ) [136,137,138].

The squamocolumnar junction (SCJ) is dynamic. The estrogenic stimulus causes the SCJ to externalize towards the ectocervix during a woman’s reproductive life [137]. The endocervical epithelium is physiologically exposed to the environment of the vagina, and undergoes a process of squamous metaplasia; the area of squamous metaplasia is called the transformation zone (TZ). The transformation of a glandular epithelium into squamous epithelium is the basis for understanding the development of squamous cell carcinoma of the cervix, as the HPV virus preferentially infects the basal cells of the cervical TZ [138,139]. The severity of cervical intraepithelial neoplasia is classified by histological abnormalities such as basal cell proliferation, nuclear enlargement, and the presence of abnormal mitotic figures [139].

The Lower Anogenital Squamous Terminology (LAST) project of the College of American Pathology and the American Society for Colposcopy and Cervical Pathology published, in 2012, a terminology to describe squamous lesions of the anogenital tract associated with HPV: low-grade squamous intraepithelial lesions (LSIL) and high-grade squamous intraepithelial lesions (HSIL) [140].

However, the Bethesda terminology is still used widely. In this system, cytologic findings are described as “squamous intraepithelial lesions (SIL)”. Histologic changes are described with the term “cervical intraepithelial neoplasia (CIN)”; CIN1 refers to a low-grade lesion with mildly atypical cellular changes in the lower third of the epithelium, CIN 2 refers to a high-grade lesion with moderately atypical cellular changes confined to the basal two-thirds of the epithelium, and CIN 3 is a high-grade lesion with severely atypical cellular changes encompassing more than two-thirds of the epithelial thickness, and includes full-thickness lesions [141,142].

From persistent infection to invasive cervix carcinoma, the carcinogenesis process takes about one or more decades in most women [143,144,145]. Cervical intraepithelial neoplasia is a dynamic condition that can progress to cervical cancer, undergo regression and viral shedding, or persist [145]. Loopik and colleagues found that the regression rates for CIN1 reached 60% in patients treated conservatively, while for CIN2 and CIN3, the rates were 55% and 28%, respectively; about 25% of CIN1 lesions persisted during the study period, as did 23% of CIN2 and 67% of CIN3; the progression rates were higher as injury severity worsened—only 14% of CIN 1 injuries progressed to CIN 2, CIN 3, or worse injuries, but 42% of CIN 2 injuries progressed [142]. Interestingly, in HSIL lesions, metalloproteases such as MMP-2 and MMP-9 are found to be more expressed when compared with LSIL lesions [108,112,146], which could be correlated with lesion progression.

## 6. HPV and Cervical Cancer

Cervical cancer is the fourth most frequently diagnosed cancer and the fourth leading cause of cancer death in women, with 342,000 deaths worldwide in 2020 [147]. The incidence and mortality rates of cervical cancer are lower in developed countries. The social and economic disparities impact cervical cancer survival even inside high-income countries, with women living in high poverty having a higher prevalence of cervical cancer [147].

Persistent HPV infection is a necessary, but not sufficient, cause for cervical cancer development [148]. Therefore, cervical cancer is considered almost entirely preventable, and HPV vaccination status (primary prophylaxis) and screening programs (secondary prophylaxis) are key indicators that impact the epidemiology of cervical cancer [147,149,150,151,152]. There are two main histologic types of cervical cancer: squamous cell carcinoma and adenocarcinoma, and both share many risk factors [153]. Studies have found that females who have not initiated sexual activity were uninfected with HPV, or have shown a very low HPV prevalence [154,155,156,157,158]. A longitudinal study carried out by Brown and colleagues published in 2005 found that about 45% of sexually active adolescent women were positive for HPV infection; the mean age of this study population was 15 years [157].

The early onset of sexual activity is a risk factor for the development of cervical cancer [159]. The development of high-grade squamous intraepithelial neoplasia and adenocarcinoma in situ (CIN2-3/AIS) was associated with a shorter interval from menarche to the first sexual intercourse [30]. Other sexually transmitted infections, such as *Chlamydia trachomatis* and HSV (herpes simplex virus), can contribute to the infection of HPV and the development of cervical lesions, including invasive carcinoma [160,161]. *C. trachomatis* can induce changes in the response to DNA damage and cell cycle control, making this infection favorable for malignant transformation [78,162,163,164,165]. Zhu and colleagues found that the coinfection of HPV and C. trachomatis promotes a higher risk of cervical cancer (OR = 4.03, 95% CI: 3.15–5.16, *p* < 0.001), both for squamous cell carcinoma and adenocarcinoma [163].

While smoking was associated with a higher risk of squamous cell carcinoma development, this association was not the case for the adenocarcinoma of the cervix [31,166,167]. Low socioeconomic status and health access determinants have also been associated with a higher incidence and prevalence of cervical cancer [147,168,169,170]. Squamous cell carcinoma accounts for nearly 75% of cervical carcinomas; however, the incidence of adenocarcinoma has increased rapidly in recent years [153,171]. It is important to know that both histological subtypes are associated with different HPV subtypes in frequency, as while HPV16 is present in almost 60% of cases of squamous cell carcinoma, HPV18 appeared in almost 40% of adenocarcinomas, as well as HPV16 [172].

The overexpression of p16 is associated with the activity of the E7 oncogene; therefore, the immunostaining of p16INK4A has emerged to differentiate HPV-dependent cervical cancer [173,174,175,176]. Furthermore, studies have shown that different metalloproteases are involved with cervical cancer. Tian and collaborators showed that MMP1 overexpression and the PPAR signaling pathway were linked to LN metastasis in cervical cancer patients [177]. MMP1 knockdown inhibited cervical cancer cell proliferation, migration, and invasion, while increasing the expression of epithelial marker E-cadherin and decreasing the expression of the metastasis-associated gene vimentin [177]. To some extent, MMP1 has a role in the regulation of cervical tumor growth and LN metastasis via EMT, and it could be a biomarker for cervical cancer LN metastasis, although more research is needed [177]. Moreover, MMP-1 and MMP-2 were shown to be highly expressed in tumor cells by immunohistochemistry, and the coexpression of these proteases in invasive carcinoma of the cervix uteri suggests a continuously rising invasive potential [108]. Furthermore, the overexpression of KLK5 is linked to the aggressiveness of cervical cancer, and may explain why traditional treatments aren’t working. As a result, KLK5 could be a good predictor of cervical cancer prognosis [178].

## 7. HPV and Vulvar Cancer

Although female genital tract cancers are commonly associated with HPV infection due to the strong association between cervical carcinoma and HPV, vulvar carcinoma is a rare genital cancer, and can arise both associated and unassociated with HPV infection [179]. Indeed, vulvar carcinomas may also be associated with chronic inflammatory lesions, such as lichen sclerosus and lichen simplex chronicus, with the latter being the most frequent [179,180,181].

HPV-induced vulvar squamous cell carcinoma (VSCC) arises from vulvar intraepithelial neoplasia, a precursor lesion [181,182,183]. The terminology used to characterize vulvar lesions has changed in recent years. The then-called ‘vulvar intraepithelial neoplasia’ (VIN) was refined to include two different lesions: usual-type VIN and differentiated VIN. The usual-type VIN is associated with HPV persistent infection, while differentiated VIN is related to chronic skin conditions [179,184].

A new terminology update was made in 2015 by The International Society for the Study of Vulvovaginal Disease (ISSVD), and vulvar lesions were divided into low-grade squamous intraepithelial lesions (LSIL), high-grade squamous intraepithelial lesions (HSIL), and differentiated VIN [184].

HSIL and invasive vulvar cancer are associated with high-risk HPV, especially HPV16, and are more often found in younger women [181]. These lesions share the same risk factors as cervical cancer, such as low economic status, immunosuppression, increasing age, smoking, and changes in sexual behavior [179]. Therefore, many patients have multifocal diseases involving the cervix, vagina, and anus [183,185,186]. The clinical manifestations of HSIL and vulvar cancer are variable, and a histological specimen is required for an accurate diagnosis [181].

Although two independent pathways have been described to explain the development of vulvar squamous cell carcinoma (VSCC), clinical and histopathological features are not sufficient to accurately determine the HPV-associated carcinoma [173,176,183]. The demonstration of the integration of HPV DNA into the genome of tumor cells would be the best way to ensure association with HPV, and hybridization tests can do this [176,182,187]. The presence of HPV-DNA alone is of questionable sufficiency to link infection to malignant transformation processes, as it may reflect an incidental finding, given the high prevalence of HPV. Therefore, the polymerase chain reaction (PCR) for HPV DNA and other commercially available tests may be insufficient to classify VSCC as HPV-associated or HPV-independent [176,182]. On the other hand, the overexpression of some proteases might be related with vulvar cancer. One study concluded that the metalloproteases MMP-2 and MMP-9 and the tissue inhibitor of matrix metalloprotease 2 (TIMP-2) overexpression could lead to higher progression rates from VIN to invasive vulvar squamous cell carcinoma [188].

## 8. HPV and Vaginal Cancer

Vaginal cancer is a rare cancer that affects the lower genital tract in women, accounting for only 1–2% of female genital tract cancers [189,190,191,192]. The vagina is a muscular structure which extends from the cervix to the hymenal ring, and is composed of a non-keratinized stratified squamous epithelium [191].

Intraepithelial lesions can precede the squamous cell carcinoma of the vagina: the precursor lesions of squamous cell carcinoma of the vagina are divided into two low-grade lesions (LSIL), in which there is evidence of HPV infection, but which can still be transient lesions and high-grade lesions (HSIL), in which there is some evidence of cellular transformation [191]. Some risk factors described for primary carcinoma of the vagina include having five or more sexual partners during one’s lifetime (OR = 3.1, 95% CI = 1.9–4.9), early age at first sexual intercourse (<17 years, OR = 2.0, 95% CI = 1.2–3.5), and current smoking at diagnosis (OR = 2.1, 95% CI = 1.4–3.1) [190,193,194].

The histopathological analysis drives the diagnosis, and the association with HPV can be made by p16 block staining [191,193]. For the diagnosis of primary vaginal cancer, it is necessary to rule out cervical or vulvar etiology, and the patient cannot have a history of these neoplasms in the last five years [190,191]. Most of the time, the vagina is affected by a local extension of neoplasms from other sites, such as the cervix, endometrium and vulva, but it can also be the site of implantation of distant metastases: mainly breast, ovarian and kidney cancer [190,195,196,197,198,199]. The primary histological type is squamous cell carcinoma, corresponding to 80–90% of cases [189,190,191]. Persistent HPV infection is believed to be associated with its development [190,191,192]. Although vaginal cancer is still not well understood, one study highlighted the potential role of KLK5, a serine protease, as a suppressor of vaginal carcinogenesis which could be used in the future as a treatment [200].

## 9. HPV and Anal Cancer

Most anal canal cancers arise around the squamocolumnar junction, and therefore can be squamous cell carcinomas or adenocarcinomas, with the former being responsible for more than 80% of anal cancers [201]. The prevalence of anal cancer has increased sharply in recent decades, especially among young men and older women, as well as the mortality associated with this neoplasm also increasing, with a more significant proportion of advanced disease [201,202,203,204,205]. This shift in the pattern of prevalence may be associated with changes in sexual behavior [204].

Current evidence points to a causal effect of HPV on the development of anal canal cancer [201,206,207,208,209,210,211,212]. As with cervical neoplasia caused by HPV, HPV-related anal neoplasia can manifest as preinvasive squamous intraepithelial lesions (SIL), which can progress from low-grade to high-grade dysplasia, and ultimately to invasive cancer [206]. Unlike the natural history of HPV in the development of cervical cancer, the behavior of HPV in anal canal malignancies is less well known [210,211,212,213]. In this context, it is essential to highlight the substantial differences in the behavior of this disease in men and women, and HIV-positive and negative patients [210,213,214]. Chunqing Lin and colleagues contributed significantly by demonstrating that HPV16 is by far the most carcinogenic type of anal cancer in both men and women, regardless of HIV status, and that HPV16 positivity increases with the lesions’ severity (high-grade dysplasia and invasive carcinoma) [213].

Patients with an intraepithelial lesion may present with bleeding or anal lesion symptoms, but the vast majority of patients are asymptomatic [215,216]. However, invasive carcinoma often presents with bleeding, tenesmus, and a palpable mass on clinical examination [206]. The definitive diagnosis is confirmed with an anatomopathological analysis of the specimen [206]. Lin and colleagues’ findings have significant repercussions for primary prevention through prophylactic HPV vaccination and secondary prevention through anal cancer screening [213].

Anal cancer screening is not a consensus among international societies [216]. Intraepithelial lesions are rare in the general population, which would not justify a universal screening program; however, screening for precancerous lesions in the higher-risk population should be considered, e.g., for HIV-positive, MSM, and immunocompromised patients, and women with a history of dysplasia or invasive cervical carcinoma [216,217,218,219,220].

## 10. HPV and Penile Cancer

Penile cancer is a rare cancer, the prevalence of which is higher in underdeveloped areas of the world [221]. It is commonly diagnosed in men over 60 [201]. HPV infection is considered a risk factor, as HPV-DNA was detected in up to 50% of cases of penile invasive carcinoma and up to 80% of intraepithelial neoplasia cases of the penis [222,223,224,225,226,227,228,229]. Although HPV infection is one of the main risk factors associated with penile cancer, other risk factors should also be considered, such as phimosis, smoking, HIV infection, and Lichen sclerosis [223,224,225,230,231].

The evaluation of p16INK4 status is helpful in order to assess the prognosis of invasive penile cancer; thus, the CDKN2A gene encodes the p16 INK4A protein, and the overexpression of this protein associated with HIV infection leads to essential changes in the cell cycle and, consequently, in the malignancy process [51,229,232,233,234]. Positive p16 INK4A is associated with a better prognosis in penile cancer [235,236,237].

## 11. HPV and Head and Neck Squamous Cell Carcinoma (HNSCC)

HNSCC is a type of cancer originating in the squamous cells of the epithelia of the following anatomical regions: the oral cavity, pharynx, larynx, nasal cavity, and salivary glands [238,239]. The incidence of this type of cancer is very high, accounting for more than 450,000 deaths each year [239,240]. It is essential to highlight that HNSCCs have different etiological factors, such as smoking, alcohol consumption, and mainly the infection by HPV viruses, especially HPV16 and 18 [241,242,243,244]. Due to the decrease in tobacco consumption worldwide, the incidence of HNSCCs, usually diagnosed in older patients, is decreasing [245]. On the other hand, the incidence of HPV16 oropharyngeal cancer is increasing in younger people, especially in North America and northern Europe, which might be associated with the 10-to-30-year latency of the virus after exposure to oral sex [246,247]. Another important aspect is the late diagnosis of HNSCCs, which directly impacts the survival rate, which was no longer than five years in 50% of the cases from 1992–1996, and has now increased to 66% from 2002 to 2006 [248,249,250]. Significantly, the treatment did not progress over the years, and cisplatin chemotherapy, radiotherapy, and surgery are the most common treatments used in the patients [248,251,252].

One of the characteristics related to cancer progression is the capacity of the cancer cells to proliferate indefinitely [11,12]. The basal layer of the epithelia can generate the other layers of this tissue, which is considered to have the potential of neoplastic cell transformation, as the basal cells renovate the epithelial tissue very quickly [253].

It has already been reported that the initial stages of HNSCCs are related to the loss of the chromosome locus 9p21 [251,254,255]. This loss is very devastating for the cells because the region encodes essential tumor suppressor genes, such as cyclin-dependent kinase inhibitors 2A (CDKN2A), CDKN2B, CDKN2B-AS1, MTAP (which encodes a vital enzyme that metabolizes polyamides), and interferon genes (IFN) [251,256,257]. The CDKN2A gene is responsible for encoding p16INK4A and p14ARF proteins, which are tumor suppressor proteins that are related with, respectively, the inactivation of kinases dependent on cyclins (CDKs) and the increase of p53 transcriptional activity through inhibition of MDM2 [257,258,259]. Mutations in the TP53 gene, which encodes the p53 protein, are associated with a worse prognosis and resistance to treatments [244]. For instance, studies have shown that mutation in the TP53 gene is related to the resistance of patients to the treatment with cisplatin [260,261].

Tobacco use is the most important risk factor for HPV-negative HNSCC development [238]. Tobacco contains approximately 5000 distinct compounds, dozens of which have been confirmed to cause cancer. The local production of cytokines, chemokines, and growth factors, which occur in tandem with inflammation, can play key roles in encouraging proliferation, angiogenesis, and, eventually, carcinogenesis [238]. Excessive alcohol intake is another important risk factor for HPV-negative HNSCC, and it has been shown to enhance carcinogenesis when combined with tobacco use [262]. Alcohol may act as a solvent for carcinogens, allowing epithelial cells to be exposed to more of them [263]. HPV infection is becoming a more common risk factor for HNSCC. Most oropharyngeal cancers (>70%) and a small percentage of malignancies in other head and neck anatomical regions are linked to HPV infection [264,265]. HPV-positive HNSCC has distinct gene expression, mutational, and immunological profiles from HPV-negative HNSCC, highlighting the disease’s specific biology [Johnson et al., 2021]. Although HPV-16 is the most common cause, other high-risk HPVs such as HPV-18, HPV-31, HPV-33, and HPV-52 are found in a small number of patients [266]. In contrast to HPV-negative HNSCC, in which the TP53 (encoding p53) gene is usually deleted or mutated, p53 is removed by the action of E6 in HPV-positive HNSCC [267].

Although there are not many studies concerning the relationship between HPV, proteases and HNSCC, it is already known that different proteases are related to HNSCCs. One study showed that serine protease TMPRSS2 expression is reduced in HNSCC, with the TP53 gene being mutated, and in HPV-negative samples, when compared with normal tissues [268]. Another study found that the E6 oncogene caused HNSCC cells to become malignant by regulating multiple pathways, and that the secretory leukocyte protease inhibitor (SLPI) could reverse the effect of the E6 oncogene on HNSCC cells, implying that the functional inhibition of E6 by SLPI could be used as a promising therapeutic strategy [269].

## 12. Conclusions

The topics discussed in this review are essential for the understanding of the complex mechanisms involved in HPV-related cancers and the action of various proteases in this context. This review highlighted the carcinogenic mechanisms involved in different HPV-related cancers, such as cervical, vulvar, vaginal, anal, and penile cancers and HNSCCs, and also highlighted the involvement of proteases with the tumor microenvironment.

## Figures and Tables

**Table 1:** Key statistics of HPV-related cancers in the world.

Population								World
Women at risk for cervical cancer (Female population aged ≥ 15 years) in millions								2869.0
Burden of cervical cancer and other HPV-related cancers								
Annual number of new cervical cancer cases								604,127
Annual number of cervical cancer deaths								341,831
Standardized incidence rates per 100,000 population:								
	Cervical Cancer	Anal Cancer	Vulva Cancer	Vaginal Cancer	Penile Cancer	Oropharyngeal Cancer	Oral cavity Cancer	Laryngeal Cancer
Men	-	0.49	-	-	0.80	1.79	5.96	3.59
Women	13.3	0.58	0.85	0.36	-	0.40	2.28	0.49
